# RAD-Seq-derived SNPs reveal no local population structure in the commercially important deep-sea queen snapper (*Etelis oculatus*) in Puerto Rico

**DOI:** 10.1007/s42995-025-00289-7

**Published:** 2025-05-12

**Authors:** María del P. González-García, Jorge R. García-Sais, Graciela García-Moliner, Nikolaos V. Schizas

**Affiliations:** 1 Museo Nacional de Ciencias Naturales, CSIC, José Gutiérrez Abascal 2, 28006 Madrid, Spain; 2 Department of Marine Sciences, University of Puerto Rico at Mayagüez, P.O. Box 9000, Mayagüez, PR 00681 USA; 3 Caribbean Fishery Management Council, 270 Muñoz Rivera Ave., Suite 401, San Juan, PR 00918-1903 USA; 4 Reef Research, Inc., P.O. Box 178, Boquerón, PR 00622 USA

**Keywords:** *Etelis oculatus*, Genetic diversity, Caribbean Fisheries, Genomics

## Abstract

The queen snapper (*Etelis oculatus* Valenciennes in Cuvier & Valenciennes, 1828) is a deep-sea snapper whose commercial importance continues to increase in the US Caribbean. However, little is known about the biology and ecology of this species. In this study, the presence of a fine-scale population structure and genetic diversity of queen snapper from Puerto Rico was assessed through 16,188 SNPs derived from the Restriction site Associated DNA Sequencing (RAD-Seq) technique. Summary statistics estimated low genetic diversity (HO = 0.333–0.264) and did not reveal population differentiation within our samples (*F*_ST_ = − 0.001–0.025). Principal component analysis and a model-based clustering method did not detect a fine-scale subpopulation structure among sampling sites, however, there was genetic variability within regions and sites. Our results have revealed comparable genetic and dispersal patterns to those observed in other shallow-water snapper species in Puerto Rico waters. It is crucial to further enhance our understanding of the ecological and biological aspect of the queen snapper to effectively manage and conserve this species as fishing pressure has been extended to deep water species in the US Caribbean.

**Supplementary Information:**

The online version contains supplementary material available at 10.1007/s42995-025-00289-7.

## Introduction

An excellent way to predict patterns in biodiversity and create good conservation strategies is by implementing population genomics (Nielsen et al. 2009). Implementing a population genomic approach can also help us make inferences about the interactions and adaptations between species and their environment (Crawford and Oleksiak 2016; Oleksiak and Rajora 2020). One crucial factor in ecology is population connectivity, defined as the successful exchange of individuals between subpopulations from a metapopulation in a given geographical area (Cowen and Sponaugle 2009). From the genomic perspective, connectivity impacts the population structure since it will tend to homogenize the variation in allele frequencies between populations (Hellberg 2009; Palumbi 2003). An important reason to study the genetic population structure of a species is that it could provide insights into how the evolutionary forces influence the species' life history traits (Weist et al. 2022). Connectivity patterns depend on the dispersal ability of the organisms, long larval durations, physical barriers, and ocean currents (Cowen and Sponaugle 2009; Selkoe et al. 2016). Many connectivity patterns are species-specific in the marine realm; therefore, there is much literature on fishes that exhibit or do not exhibit population structure, making them the most studied taxon on this topic (e.g. Beltrán et al. 2017; Bryan-Brown et al. 2017; Tovar Verba et al. 2023). In addition, genomic data has been used to assess the population structure of many commercial important marine fishes to address many questions related to evolution and phylogenetic relationships, geographical distribution and dispersal, and conservation management (Bors et al. 2019; Herrera et al. 2015; Sherman et al. 2020). It has been shown that every studied system is different and that the results should not be extrapolated between species. Especially when deep-sea habitats are been studied, they could yield unique dispersal and demographic patterns for fishes (Andrews et al. 2020; Baco et al. 2016). Studying deep-sea species with limited ecological or biological data can present additional challenges in choosing the appropriate sampling methodology. Due to the logistic complications of studying demographic parameters using direct methods with deep sea fishes, implementing population genomics methods is a helpful solution.

Some of the fisheries in the Caribbean (e.g. several species of groupers and snappers) have varying levels of consistent or no available data, and many of these species are under threatened status (Galindo-Cortes et al. 2019; Salas et al. 2011; Tonioli and Agar 2011). Even though most of the fisheries are artisanal, there are already plenty of cases where stocks have been overfished or depleted (de Oliveira-Leis et al. 2019; Kadison et al. 2017). For example, in Puerto Rico several reef fish stocks collapsed during the 1980’s, such as Nassau grouper (*Epinephelus striatus*), goliath grouper (*Epinephelus itajara*) and red hind (*Epinephelus guttatus*) (Matos-Caraballo 2009). Most of these species were exploited during spawning aggregations, which led to the extirpation of some aggregations and the commercial extinction of the Nassau grouper during the 1990’s (Schärer-Umpierre et al. 2014). During the late 1990’s and early 2000’s, an increase in the landing pounds was observed in three deep-water snapper species from the Snapper Unit 1 composed of the silk snapper (*Lutjanus vivanus*), the blackfin snapper (*Lutjanus buccanella*), and vermilion snapper (*Rhomboplites aurorubens*) (SEDAR4 2003; NOAA 2011; Tonioli and Agar 2011). Snapper Unit 1 indicates a fishery in the early stages of overfishing in Puerto Rico (NOAA 2011; Tonioli and Agar 2011). Possibly due to this, an increase in queen snapper landings was recorded and it reached up to 10% of the total landing pounds per year (Matos-Caraballo 2012). Nevertheless, quantifying the exact historical demand of the queen snapper is futile since landings of this species were commonly misidentified as silk snapper; this taxonomic error has reduced the value of previous reports (Matos-Caraballo 2000; Cumming and Matos-Caraballo 2003). Currently, the deep water fishery of queen snapper is not regulated besides the requirement of a special commercial fishing license for fishing this species. Around 65 fishers in Puerto Rico have currently (2022) this license (García-Sais, personal observation). Basic biological knowledge of the demography and regional population structure of this species remains unknown (SEDAR4 2003; SEDAR26 2011; Tonioli and Agar 2011; Crabtree 2019).

The queen snapper (*Etelis oculatus* Valenciennes in Cuvier and Valenciennes 1828) is the only species from the *Etelis* genus in the Atlantic believed to have diverged around 0.5 mya from *E. coruscans* from the Indo-Pacific (Andrews et al. 2016; Leis and Lee 1994). The geographical distribution is restricted to the tropical western Atlantic Ocean, from North Carolina to Brazil; it is most abundant in the Bahamas and Antilles (Allen 1985). It has pink to red perciform body; with a small head, a distinct large eye, and a short snout (SEDAR4 2003). The maximum total length could reach about 60 cm but is more commonly observed at 52 cm (Allen 1985). A study on age estimation has concluded that queen snapper can live for over 40 years (Overly and Shervette 2023). This species usually inhabits rocky bottom habitats of 100–500 m, with the deepest record at 539 m near Caja de Muertos Island observed on an Okeanos Expedition in 2018 off the south coast of Puerto Rico (Gobert et al. 2005). The larvae of this species can be found from 0 to 100 m in depth and can survive in the water column for up to 26 days. They have also been observed in the gyres of the Sargasso Sea (D’Alessandro et al. 2010; Leis and Lee 1994). Since the larvae can survive in far offshore waters and have an extended pelagic phase, the dispersal potential of the queen snapper should be significantly large. Until today, only three studies have addressed morphological data, age estimation, reproduction, and diet composition of queen snapper on Puerto Rico (Overly and Shervette 2023; Rosario et al. 2006; Williams et al. 2024).

There is an ongoing study from the Caribbean Fishery Management Council (CFMC) in Puerto Rico to characterize the benthic habitats, feeding habits, and other life history strategies from deep water stocks of commercially important species (García-Sais, unpublished data). Within this project, the current study proposed to conduct a population genomic analysis of queen snappers, the most targeted snapper species from the Snapper Unit 2. Our objective was to estimate the standing genetic diversity, population structure, and geographic connectivity of *E. oculatus* around Puerto Rico. Through SNP-derived genotypes from Restriction site Associated DNA Sequencing (RAD-Seq), we tested for the presence of fine-scale population structure in Puerto Rico. The RAD-Seq de novo technique is widely used in studies to assess population structure given its ability to genotype thousands of markers for population-specific variants (Peterson et al. 2012). We hypothesized that we will detect small but significant population genetic differences among Puerto Rico queen snapper based on the distance between the sampling areas and the prevailing surface currents in Puerto Rico. The presence of genetic differentiation would indicate that the relatively high larval dispersal potential may not be a good predictor of realized dispersal and connectivity of queen snappers inhabiting Puerto Rico waters and the absence of population structure would indicate high levels of population connectivity around the island of Puerto Rico.

## Materials and methods

### Sample collection, DNA extractions, RAD-seq library creation and sequencing

Queen snappers were sampled during ten fishing trips with four different fishers using longlines with electric reels. The geographic coordinates and depths were recorded in all sampling stations, but the coordinates will not be revealed to protect the fisher’s fishing sites. Upon landings of the queen snapper, fork length and weight were recorded. All specimens remained in ice or frozen until processed at the Isla Magüeyes Marine Research Station. At the laboratory, tissue samples from the caudal fin muscle and caudal fin clip were preserved in 100% ethanol for each specimen following sterile procedures. All tissue samples were stored in a − 20 °C freezer until the DNA extraction step. The DNA extractions were carried out using the Qiagen DNeasy 96 Blood and Tissue Kit (Qiagen, Germany) following the manufacturer’s protocol. DNA extractions were quantified with the Qubit 2.0 Fluorometer (Life Technologies, Carlsbad, California, US) to confirm that each sample had a DNA concentration of at least 25 ng/μL before sending them to the Genomic Sequencing Facilities for library preparation and sequencing. Once all the samples met the criteria (quantity and quality), DNA extractions were sent to Admera Health (South Plainfield, NJ) for RAD-seq library preparation using *SbfI* as the digestion enzyme. The same Genomic Facility carried out 2 × 150 bp paired-end sequencing with Nova Seq S4 targeting 3.7 M total reads per individual, around 1.85 M in each direction.

### Choosing the correct parameters for denovo.pl program

The quality of the raw reads was checked using FastQC. The *process_radtags* script from the pipeline *Stacks* v. 2.6 was used for demultiplexing, cleaning, and discarding the low-quality reads (Catchen et al. 2013). Given the lack of previously published genomic data on the queen snapper, we followed the parameter optimization protocol from Rivera-Colón and Catchen (2022) to obtain the highest number of polymorphic loci in 80% of the samples. A subsample of 12 queen snappers was selected randomly for this optimization protocol. Once the protocol was run, the *denovo_map.pl* program was used with the following parameters: -M 2 (*ustacks*) and -n 2 (*cstacks*). PCR duplicates were discarded using *gstacks* and the parameter–min-samples-per-pop was set at 0.80 in the *populations* program which retained 80% of the loci found in all samples per population. A whitelist with specific SNPs was created to rerun the *populations* program with the purpose of only processing the loci from the whitelist in the analysis. The whitelist was created after filtering out the loci with missing call rates of 0.2 and an MAF of 0.05 with Plink v. 1.9 (Purcell et al. 2007). The *populations* program was rerun twice to only include the loci present in the whitelist, changing the population map per run. One population map was based on assigning the samples to locations according to the cardinal points (NWSE) and on the another map the samples were noted according to the location of collection.

### Population structure and genetic diversity

Principal Component Analysis (PCA) and Discriminant Principal Component Analysis (DAPC) were performed using the VCF output generated from Stacks. To exclude SNPs under positive selection, BayeScan (v 2.1) was used for the detection of outlier loci (Foll and Gaggiotti 2008). All the parameters were left as default, except the prior odds, which were increased to 100 due to the large number of SNPs. VCF files were converted to genind and genlight objects using vcfR package 1.14.0 in R (Knaus and Grünwald 2017). Other packages used for the analysis and visualization of the PCA and population membership probabilities obtained from the DAPC were: adegenet v 2.1.10, ape v 5.5, poppr v 2.9.3 and RColorBrewer v 1.1-3 (Jombart 2008; Paradiset al. 2004; Kamvar et al. 2014; Neuwirth and Brewer 2014). The *boot.ppfst* function from *hierfstat* was used to calculate *F*_ST_ and confidence intervals (95% CI) with 10,000 bootstraps (Goudet and Jombart 2015). GenoDive v 3.0.6 was used to calculate AMOVA assuming the Infinite Allele Model with a number of 20,000 permutations (Weir and Cockerham 1984; Meirmans 2020). Nucleotide diversity (*π*) was obtained from the *populations* output files. For inferring population structure with Bayesian clustering software STRUCTURE v 2.3.4 using the out file from the populations run from the sites, the scripts from StrAuto v1.0 were used (Pritchard et al. 2000; Chhatre and Emerson 2017). The K values were set from 1 to 5, with 10 interactions per K value. There was a total of 10,000 burn-in interactions followed by 100,000 MCMC steps. To assess the K values results and to visualize the out files from STRUCTURE, the program StructureSelector was used (Li and Liu 2018). Using the Evanno plot generated in StructureSelector, the cluster (K) with the highest Delta K was selected as the best fit for the data (Evanno et al. 2005). For the visualization of the clustering analysis using *K* values = 2–4, the program Clumpak was utilized (Kopelman et al. 2015). Contemporary gene flow among sites was inferred using BayesAss v 3.0.4 (Mussmann et al. 2019; Wilson and Rannala 2003). The Bayesian run was performed using 10^6^ MCMC iterations, a burn-in period of 10^5^, and a sampling interval of 100 (Wilson and Rannala 2003). Following the user’s manual recommendation regarding the acceptance rates; the mixing parameters deltA, deltaF, and deltaM were set to 1, 0.8, and 10^–3^, respectively (Wilson and Rannala 2003).

## Results

### Sample collection, DNA extractions, RAD-seq library creation and sequencing

**Fig.1:**
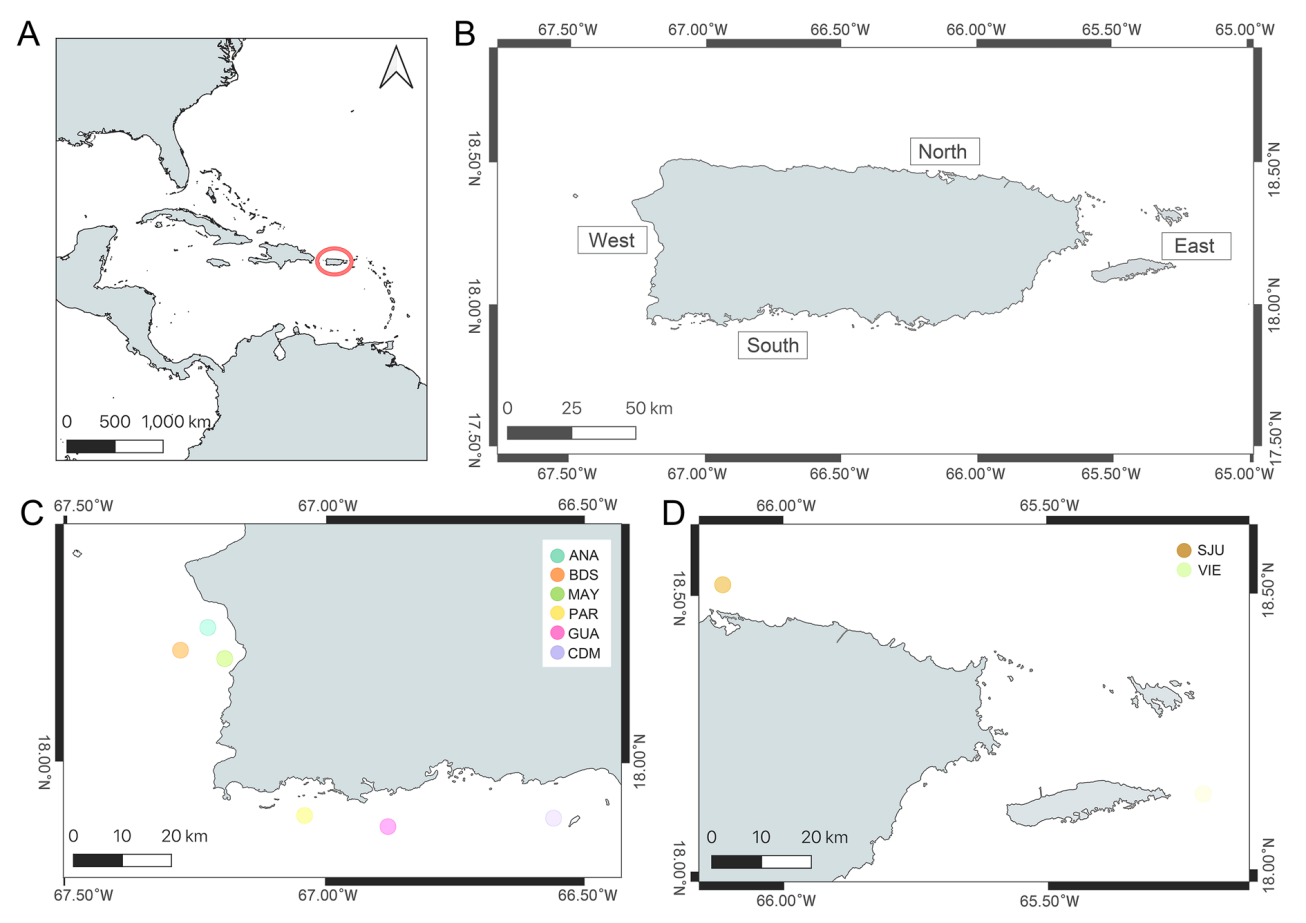
Map of the study area. **A** Location of Puerto Rico in the greater Caribbean. **B** Representation of the regions where the queen snappers (*E. oculatus*) were fished according to the regions. **C** and **D** Dots indicate approximate fishing sites of this study

A total of 37 queen snappers were fished at eight sampling sites [Mayagüez (MAY): 2, Añasco (ANA): 4, Bajo de Sico (BDS): 3, Caja de Muertos (CDM): 3, La Parguera (PAR): 5, Guánica (GUA): 3, San Juan (SJU): 6, and Vieques (VIE): 11] between 2020 and 2022 (Table S1). The sampling sites were grouped according to their cardinal points in Puerto Rico, and from now on, will be referred to as regions (Fig. 1). The specimens were fished at a depth range of 269 to 456 m, averaging 329 m. The fork lengths of the queen snapper ranged from 25.4 to 49 cm (avg. 35.5 cm) and the weights were from 0.22 to 1.67 kg (avg. 0.670 kg). DNA extraction concentrations ranged from 27.2 up to 110 ng/μL (avg. 81.26 ng/μL). RAD-Seq libraries were created for each of the 37 queen snappers, with concentrations that ranged 9.9 up to 171.0 ng/μL (avg. 116.2 ng/μL) per library. A total of 295,098,020 raw reads were obtained, the average was 7,975,622 reads per library.

### Choosing the correct parameters for denovo.pl program

After removing low-quality reads with *process_radtags*, a total of 293,845,969 reads were left for the analysis with an average of 7,941,782 reads per sample; the percentage retained was 99.58% of the raw reads (Table S2). Using the subsamples for the optimization protocol, the retained number of loci after the protocol ranged from 31,180 to 48,953 per sample. The parameters that provided the highest number of polymorphic loci in 80% of the samples were M 2 and n 2 (Table S3). After running *denovo_map.pl* with all 37 samples, a total of 226,300 loci were built in *gstacks*. The coverage per sample ranged from 16.9 × to 36.9 × with an average of 26.8 ×, with a mean number of sites per locus of 382.3 bp (Table S4). A whitelist containing 16,188 SNPs was obtained by running the Plink program filtering out the loci with 20% of missing data and with a minor allele frequency (MAF) of 0.05. Once the *populations* program was rerun with only the SNPs from the whitelist and the constraints specified, a total of 16,188 SNPs remained for the rest of the analysis. The number of loci per individual sample ranged from 15,678 to 16,015, with an average of 15,899 loci per sample. No sample had a missing loci frequency higher than 3.15%. No loci were identified under potential positive selection (Fig. S1).

### Population structure and genetic diversity

Most of the measurements of genetic diversity were consistent between all regions and sites (Table 1). The number of alleles and the effective number of alleles were lower in the site`s measurements than in the regions. The average genetic diversity (HE) was 0.275 for the regions and 0.274 for sites. The mean observed heterozygosity (HO) was 0.284 and 0.283, respectively. For the regions, the north had the highest HO (0.301) and the south had the lowest (0.264). At the sites, the highest was MAY (0.333) and the lowest PAR site (0.264). The inbreeding coefficient (GIS) was highest in the south (0.033) and the GUA site (0.038). GIS was lowest in the west regions (− 0.061) and the MAY site (− 0.216).

**Table 1:** Summary indices of genetic diversity per region or site based on the resulting 16,188 SNPs

Region/Site	No. alleles	Effective no. alleles	*H*_o_	*H*_*E*_	*G*_IS_	*π*
SOUTH	1.929	1.413	0.264	0.274	0.033	0.246
WEST	1.888	1.416	0.291	0.274	− 0.061	0.245
NORTH	1.793	1.413	0.301	0.276	− 0.09	0.247
EAST	1.936	1.418	0.281	0.276	− 0.017	0.247
BDS	1.615	1.38	0.274	0.273	− 0.004	0.246
MAY	1.535	1.385	0.333	0.274	− 0.216	0.245
ANA	1.696	1.394	0.282	0.273	− 0.031	0.243
PAR	1.754	1.397	0.264	0.273	0.033	0.245
GUA	1.612	1.38	0.265	0.276	0.038	0.260
CDM	1.612	1.376	0.265	0.273	0.03	0.244
SJU	1.793	1.413	0.301	0.276	− 0.09	0.249
VIE	1.936	1.418	0.281	0.276	− 0.017	0.249

*GUA* Guánica, *CDM* Caja de Muertos, *PAR* La Parguera, *BDS* Bajo de Sico, *MAY* Mayagüez, *ANA* Añasco, *SJU* San Juan, *VIE* Vieques

**Fig.2:**
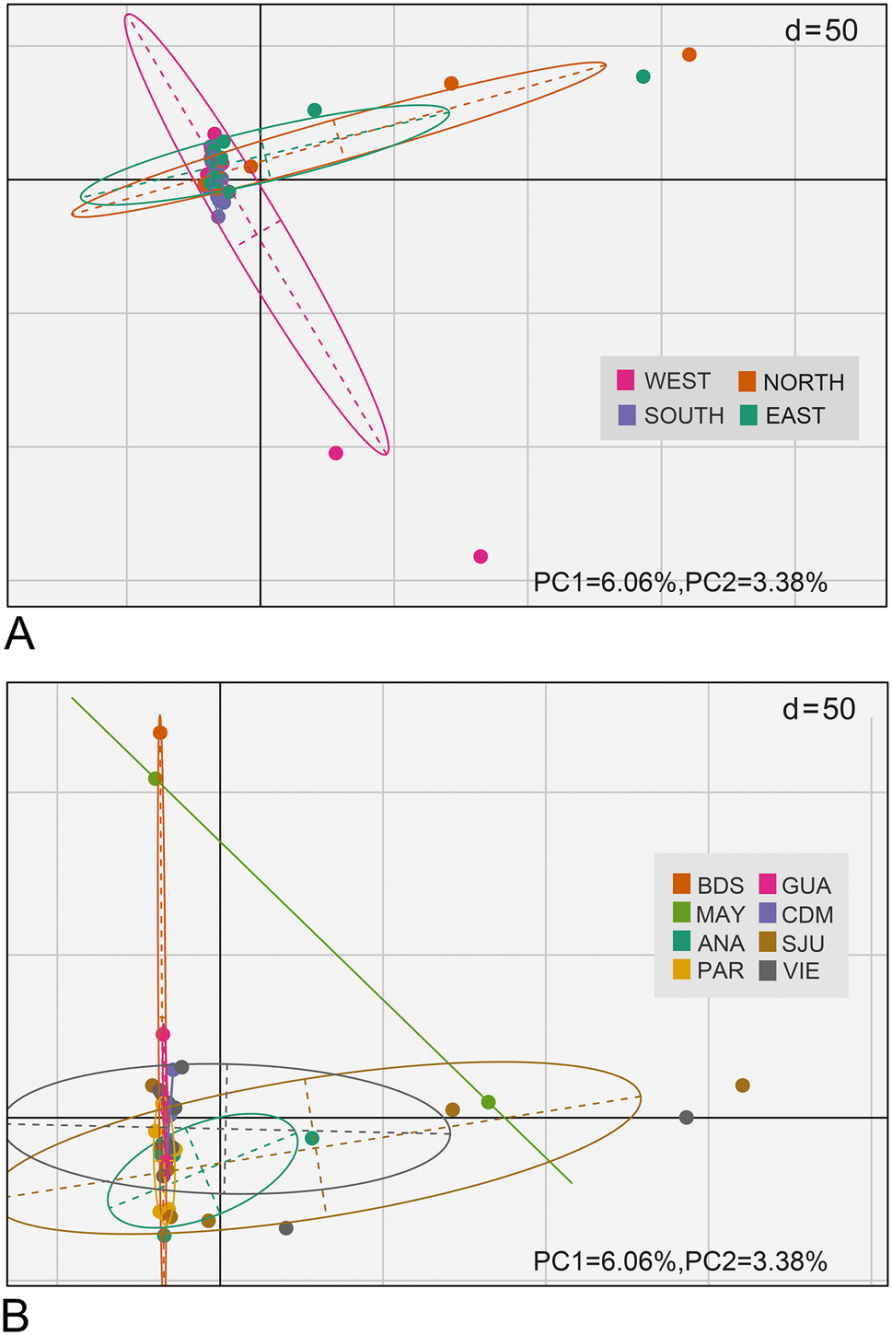
Principal component analysis (PCA) based on 16,188 SNPs, showing genetic variation among the 37 queen snappers (*E. oculatus*)) when grouped by **A** regions and **B** sites

**Fig.3:**
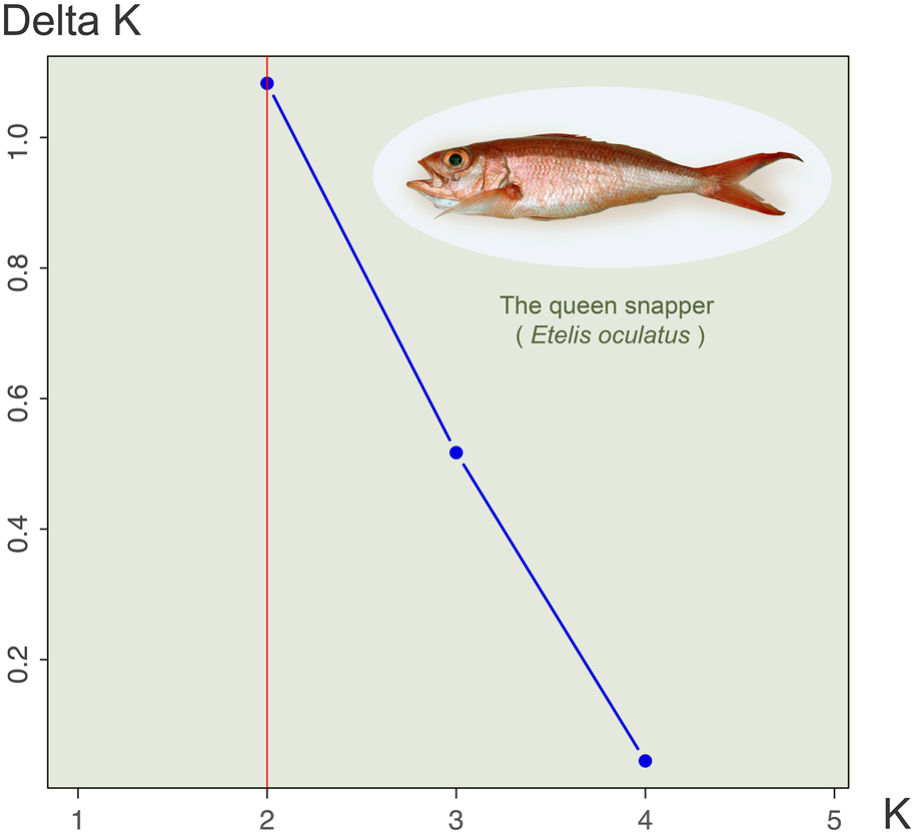
Evanno plot from StructureSelector (Evanno et al. 2005) of Delta K values change due to the cluster inference (*K* = 1–5). Each value of K was tested with 10 replicate simulations

**Fig.4:**
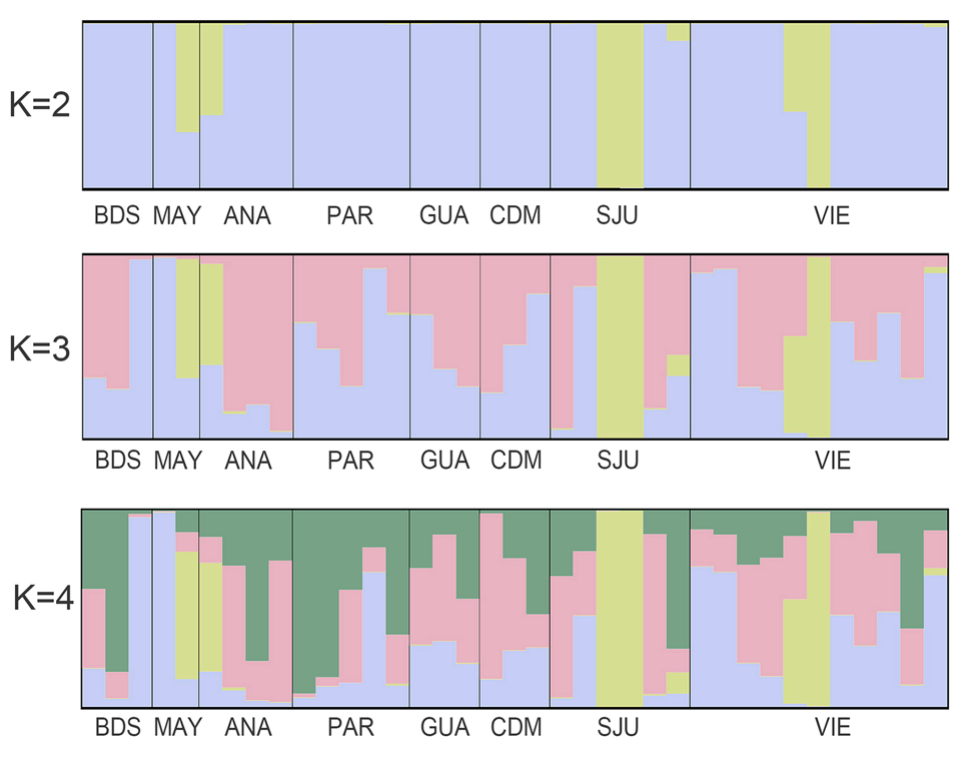
Population structure analysis using STRUCTURE from the 16,188 SNPs for queen snapper (*E. oculatus*). Each line represents an individual and the estimated proportions (y-axis) from each inferred cluster (K) of the sampling sites (x-axis)

**Fig.5:**
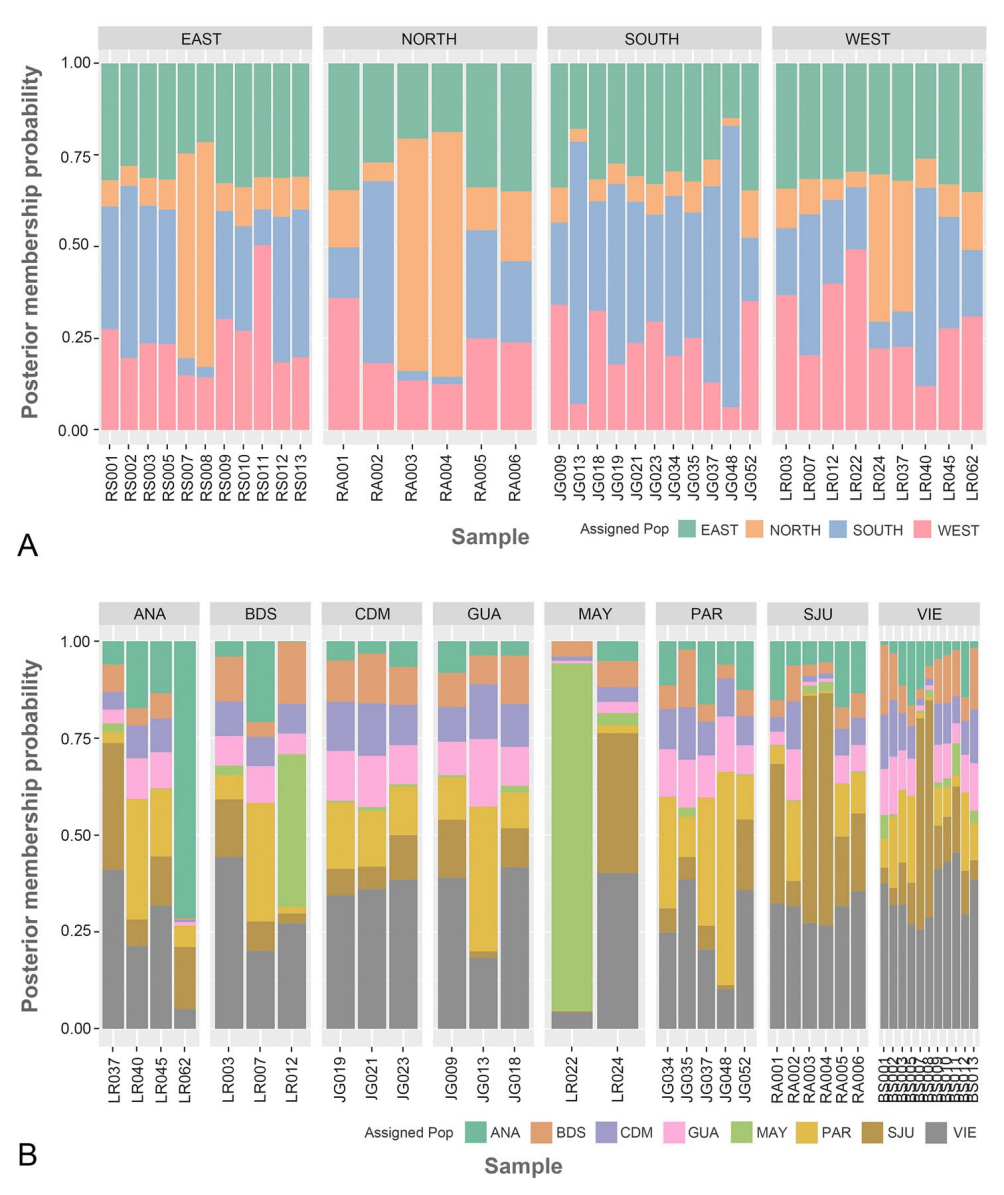
Population membership probabilities from the Discriminant Principal Component Analysis (DPCA) using 16,188 SNPs, grouping individuals by **A** regions and **B** sites. Each line represents an individual, with the y-axis indicating the assigned population proportions

Pairwise *F*_ST_ comparisons were significant between three regions and between eight sites (Tables 2 and 3). Based on these values, in regions *F*_ST_ ranged from − 0.00234 to 0.00709 and in sites from − 0.02776 to 0.01464 (Tables 2 and 3). According to the AMOVA test, most of the genetic variability occurred within individuals in both regions and sites models (Table 4). In the regions PCA, individual fish from the west and east regions that are separate from their own localities and the south region (Fig. 2A). All regions showed genetic variability within the groups, except the south where all samples were grouped in the PCA (Fig. 2A). In sites PCA, the samples from MAY separate from the rest of the sites (Fig. 2B. Individuals from BDS, SJU and VIE exhibited genetic variability both within their respective sites and among individuals from other sites (Fig. 2B). Following the Evanno et al. (2005) protocol, the highest Delta *K* was obtained when *K* value = 2; suggesting that the queen snappers in this study may be composed of two sub-populations (Fig. 3). Nevertheless, the clustering level of *K* = 1 yielded the largest LnP(K) (Fig. S2). Most individuals belong to one genetic cluster when *K* = 2, but there are six individuals that are mixed by the two genetic clusters (Fig. 4). In addition, two queen snappers from SJU and one from VIE consist of the second cluster (Fig. 4). These unique individuals did not show any admixture with the other genetic clusters from the analysis, and this observation remained consistent throughout the different *K* values (2–4) (Fig. 4). From the DPCA analysis, the probability membership frequencies from the east region and VIE site remained consistent in all other regions and sites (Fig. 5). One individual from ANA and another from MAY distinctly differentiated themselves from all other members of their sites. While collected from the SJU and VIE sites, two individual fish showed notable membership probability frequencies from the SJU-assigned population site (Fig. 5B). The inferred results from BA3SNP for the migration rates between sites indicate a contemporary gene flow from VIE to the other sites (Table S5). The migration rates from VIE to the rest of sites are consistent, maintaining a rate from 10 to 16% in each site (Table S5). VIE had the highest self-recruitment rate of 85.9% compared to the other sites (69.1–69.9%) (Table S5).

**Table 2:** Estimated 95% confidence intervals (CI) of pairwise *F*_ST_ comparisons among four regions around Puerto Rico, based on 1000 bootstraps

	SOUTH	WEST	NORTH	EAST
SOUTH	–	**0.00411**	**0.00479**	**0.00274**
WEST	**0.00139**	–	**0.00709**	**0.00417**
NORTH	**0.00128**	**0.00328**	–	0.00105
EAST	**0.00022**	**0.00146**	− 0.00234	–

The upper limit (97.5%) is above diagonal, while the lower limit below the diagonal (2.75%). Significance is shown in bold

**Table 3:** Estimated 95% confidence interval (CI) of pairwise *F*_ST_ comparisons among sampling locations, based on 1000 bootstraps

	BDS	MAY	ANA	PAR	GUA	CDM	SJU	VIE
BDS	–	− 0.01613	0.00577	0.00308	0.00170	0.00624	**0.01364**	0.00388
MAY	− 0.02776	–	− 0.01132	− 0.00511	− 0.00349	− 0.00144	0.00098	− 0.01024
ANA	− 0.00172	− 0.02204	–	0.00447	0.00510	0.00782	**0.00684**	0.00282
PAR	− 0.00390	− 0.01552	− 0.00161	–	0.00126	0.00710	**0.00930**	**0.00537**
GUA	− 0.00735	− 0.01576	− 0.00294	− 0.00603	–	0.00378	**0.01464**	**0.00640**
CDM	− 0.00340	− 0.01480	− 0.00017	− 0.00053	− 0.00584	–	**0.01457**	**0.00726**
SJU	**0.00719**	− 0.00784	**0.00167**	**0.00411**	**0.00788**	**0.00766**	–	0.00098
VIE	− 0.00139	− 0.01827	− 0.00162	**0.00132**	**0.00066**	**0.00133**	− 0.00225	–

The upper limit (97.5%) is above the diagonal, while the lower limit below the diagonal (2.75%). Significance is shown in bold

*GUA* Guánica, *CDM* Caja de Muertos, *PAR* La Parguera, *BDS* Bajo de Sico, *MAY* Mayagüez, *ANA* Añasco, *SJU* San Juan, *VIE* Vieques

**Table 4:** AMOVA results in GenoDive v 3.0.6

Source of variation	Nested in	%var	*F*-stat	*F* value	Std. Dev.	c.i. 2.5%	c.i. 97.5%	*p* value	*F*' value
AMOVA 1									
Within individual	–	1.021	*F*_IT_	− 0.021	0.002	− 0.025	− 0.018	–	–
Among individual	Population	− 0.024	*F*_IS_	− 0.024	0.002	− 0.027	− 0.02	1	–
Among population	–	0.002	*F*_ST_	0.002	0	0.002	0.003	0.001	0.003
AMOVA 2									
Within individual	–	1.022	*F*_IT_	− 0.022	0.002	− 0.025	− 0.019	–	–
Among individual	Population	− 0.023	*F*_IS_	− 0.023	0.002	− 0.027	− 0.02	1	–
Among population	–	0.001	*F*_ST_	0.001	0.001	0	0.002	0.031	0.002

The AMOVA 1 test is using SNPs at the region level and AMOVA 2 test is using SNPs at the sites level from the 37 queen snappers (*E. oculatus*)

*% var* % variability, *Std. Dev.* Standard Deviation, *c.i.* Confidence Intervals

## Discussion

The average fork length and weight of the 37 queen snappers fished for this study was slightly smaller than those reported from previous studies of *E. oculatus* from the west coast of Puerto Rico (Rosario et al. 2006; Williams et al. 2022). Both studies reported fork lengths longer than 70 cm (Rosario et al. 2006; Williams et al. 2022). The biggest fork length in the current study was 49 cm. Fork length differences between the current study and the aforementioned studies may be related to the sampling locations, seasonality, and/or small sample size (Williams et al. 2022). A total of 16,188 SNPs were identified from our 37 specimens of queen snapper. This is a higher number of SNPs compared to other studies using RAD-Seq in snappers or other marine fishes in the Caribbean (Beltrán et al. 2017; Bors et al. 2019; Sherman et al. 2020; Willis et al. 2022). Analyses of 1000’s of SNPs can illuminate the differences between individuals at the nucleotide level, the highest possible level of genetic resolution (Kool et al. 2013). Data containing a large number of SNPs can provide a higher resolution compared to other genomic markers (e.g. microsatellites) that may lack the ability to detect population differentiation effectively (Vineesh et al. 2023). The power of high-density SNP data is exemplified by various studies finding no significant differences in the inferred genetic estimations from a few individuals (*n* = 1–4) per population vs. all sampled individuals per population (Beltrán et al. 2017; Nazareno et al. 2017; Qu et al. 2020).

The pairwise *F*_ST_ values from this study are low and did not reveal the population structure for queen snappers of Puerto Rico. The higher *F*_ST_ values were observed for the north region and the SJU site. Only two queen snappers from the SJU site were separated from the rest of the PCA site samples, but they were not identified as a unique genetic cluster. Most of the variation from the PCA was within the groups of the regions and sites, concurring with the AMOVA results that the highest percent of variability was observed within the individuals (Table 4). Our low levels of observed heterozygosity and nucleotide diversity could also explain our low *F*_ST_ values in the queen snapper used in this study. Previous studies of genetic diversity in shallow water snappers of the Caribbean Sea and the Gulf of Mexico have found higher heterozygosity values, ranging from 0.583 to 0.631 (Carson et al. 2011; Saillant et al. 2012; Hollenbeck et al. 2015; Rosado Nic et al. 2020). The inbreeding coefficient values in our study were almost zero in all regions and sites, suggesting little or non-inbreeding within the individuals from our sampled locations.

There are two biogeographic breaks proposed for the connectivity of larval exchange within the Caribbean. One in the eastern Caribbean (near the Mona Passage down to Colombia) and the other one located in the Nicaragua Rise (near Jamaica to México) (Cowen et al. 2006; Hernández et al. 2023). Studies in the Caribbean Sea have suggested the possibility of a subdivision in different demographic stocks of shallow water snappers (mutton snapper: Carson et al. 2011; yellowtail snapper: Saillant et al. 2012). A study using mtDNA and microsatellites found differences in the effective population size of mutton snapper within two sites in Puerto Rico, suggesting that the west and east side of the island could host different demographic stocks (Carson et al. 2011). In our STRUCTURE results, we had similar observations due to the presence of individuals who did not show any admixture and presented different allele frequencies in SJU and VIE sites. Even though genetic similarities between the west and east sampling regions were noted (low *F*_ST_ values), our PCAs analysis showed differentiation in the queen snappers from the west region of Puerto Rico, particularly at the MAY site. Our findings from the west region and sites are similar to the results from Willis et al. (2022), who observed variations in the spatiotemporal genetic diversity from juvenile silk snapper (*Lutjanus vivanus*) individuals from the west coast of Puerto Rico, suggesting that the off-shelf BDS seamount (abbreviated as BJC in their study) has a different local recruitment in comparison to the recruitment at sites that are located on-shelf and that, in general, the recruitment processes that happens in the west coast is more influenced by semi-independent spawner units with strong spatiotemporal variations (Willis et al. 2022).

Our study sites from the west coast are located in the Mona Passage, an area with distinctive oceanographic and geomorphological features that could serve as a barrier for larval dispersal (Baums et al. 2006). Some of the features within this area are seasonal small-scale eddies, seasonal northward surface flow, four seamounts (including BDS), and the Mona, Monito, and Desecheo islands (Baums et al. 2006; García-Sais et al. 2007). Mona Passage has been shown to be a genetic barrier for fishes and other organisms within the Caribbean (Taylor and Hellberg 2003; Baums et al. 2006; Beltrán et al. 2017). Furthermore, the locality on the west coast is on the island’s leeward side where most recruitment may be local (Swearer et al. 1999). It is not known if the queen snapper could exhibit site fidelity or form seasonal spawning aggregations as known for other snapper species (Ojeda-Serrano et al. 2007; Biggs and Nemeth 2016). A fine-scale study of the horizontal and vertical movements of *Etelis coruscans*, tracked a third of the sampled individuals for almost 35 days (Okuyama et al. 2019). The fish that remained at the study site (sea bank in Japan) moved 2.0 km horizontally and 50 m vertically on a daily basis. The sample size was too small to validate that *E. coruscans* showed site fidelity, but small sample sizes are to be expected due to the complexities of sampling organisms from the deep sea. Nevertheless, the authors suggested that the fish could have remained near the bank (Okuyama et al. 2019). All these previously mentioned factors could create complex dynamics in the western Puerto Rico shelf between the larvae retention, recruitment, and adult site fidelity for the queen snapper populations.

A simulation study with fish larvae represented as particles in coastal circulations models of the US Caribbean to predict spawning aggregations (Xu 2022) showed that larval dispersal and the successful arrival rate of mutton snapper tend to be higher on the south coast of PR. It also showed that Vieques is a stepping stone for larvae from the mutton snapper spawning aggregation in St. Croix (Xu 2022). Though mutton and queen snappers share similar pelagic larval durations (PLDs), they have different depth distributions. This could suggest dissimilar pathways for their early-stage larvae due to the difference in habitat depth (D’Alessandro et al. 2010). Fish larvae can disperse further when they are near the surface than larvae that remain at depth (Gary et al. 2020). Nevertheless, we are proposing a similar dispersal pattern for the queen snapper as observed in Xu (2022), where the south receives larvae originated from the east. The estimated migration rates in this study, suggest an eastward dispersal pattern that spreads from Vieques to the rest of the sites. This dispersal pattern could be explained by the *F*_ST_ values, the distinct clustering of the north region in the PCA analysis, and the consistency in the membership probabilities from the DPCA analysis from the east region and Vieques site through the different sites in Puerto Rico.

This study marks the first time that SNP-derived genotypes from RAD-Seq have been used to characterize the genetic diversity of *E. oculatus* in Puerto Rico. The results of genetic diversity and population structure analysis based on the 37 deep-sea queen snappers are showing similar patterns as other shallow-water snapper species in Puerto Rico. In addition, observations of the consistency of east and VIE frequencies throughout the whole data set coincided with the prediction of the surface current models that have been previously proposed for Puerto Rico. The findings demonstrate the effectiveness of the RAD-Seq technique for population structure studies, even with a smaller sample size compared to other regional fish studies. We conclude that there is one genetic cluster of queen snapper present within Puerto Rico waters. However, it is inconclusive to assert if this genetic cluster is only present in Puerto Rico or have a wider distribution in other waters within the Caribbean. Subsequent studies can build on the current results by expanding their scope with additional Caribbean sampling sites, especially on the west coast of Puerto Rico and the east coast of Dominican Republic to encompass samples from across the Mona Passage. More information on the life history strategies and habitat characterization of the queen snapper is critically needed for the management of the species.

## Supplementary Information

Below is the link to the electronic supplementary material.

Supplementary file1 (DOCX 1576 KB)

## Data Availability

Raw Illumina reads for all individuals are available in the National Center for Biotechnology Information Sequence Read Archive (https://www.ncbi.nlm.nih.gov/sra; BioProject PRJNA1064466). All other data analysed have been included in the manuscript and supplementary information file.
