# Supplementary material for: RAD-Seq-derived SNPs reveal no local population structure in the commercially important deep-sea queen snapper (Etelis oculatus) in Puerto Rico
Source: Mar Life Sci Technol. 2025 May 12;7(3):594–605. doi: 10.1007/s42995-025-00289-7 (PMC12413376; doi:10.1007/s42995-025-00289-7)
Supplement: Supplementary file 1 — Supplementary file1 (DOCX 1576 KB) [file 42995_2025_289_MOESM1_ESM.docx]

**RAD-Seq derived SNPs reveal no local population structure in the commercially important deep-sea queen snapper (*Etelis oculatus*) in Puerto Rico**

María del P. González-García ^1^*, Jorge R. García-Sais^2,3^, Graciela García-Moliner^2^, Nikolaos V. Schizas^1^*

^1^University of Puerto Rico at Mayagüez, Department of Marine Sciences, P.O. Box 9000, Mayagüez, Puerto Rico 00681, USA.

^2^Caribbean Fishery Management Council, 270 Muñoz Rivera Ave., Suite 401, San Juan, Puerto Rico 00918-1903

^3^ Reef Research, Inc., P.O. Box 178, Boquerón, Puerto Rico 00622

*Corresponding Authors: maria.gonzalez67@upr.edu; nikolaos.schizas@upr.edu

| **Sample ID** | **Fork length (cm)** | **Weight (kg)** | **Sampling Date (m/d/y)** | **Site** | **Sample ID** | **Fork length (cm)** | **Weight (kg)** | **Sampling Date (m/d/y)** | **Site** |
| --- | --- | --- | --- | --- | --- | --- | --- | --- | --- |
|  |  |  |  |  |  |  |  |  |  |
| JG009 | 29 | 0.35 | 12/1/20 | GUA | LR062 | 49 | 1.6 | 8/19/20 | ANA |
| JG013 | 42.8 | 1.67 | 12/1/20 | GUA | RA001 | 36.5 | 0.72 | 9/22/21 | SJU |
| JG018 | 35 | 0.7 | 12/1/20 | GUA | RA002 | 27 | 0.35 | 9/22/21 | SJU |
| JG019 | 29 | 0.44 | 12/2/20 | CDM | RA003 | 30 | - | 9/23/21 | SJU |
| JG021 | 31 | 0.46 | 12/2/20 | CDM | RA004 | 39.8 | - | 9/23/21 | SJU |
| JG023 | 43 | 1 | 12/2/20 | CDM | RA005 | 46 | - | 9/23/21 | SJU |
| JG034 | 30.2 | 0.41 | 12/3/20 | PAR | RA006 | 37 | - | 9/23/21 | SJU |
| JG035 | 43 | 1.16 | 12/3/20 | PAR | RS001 | 34.5 | 0.46 | 4/22/22 | VIE |
| JG037 | 26 | 0.24 | 12/3/20 | PAR | RS002 | 27.2 | 0.22 | 4/23/22 | VIE |
| JG048 | 24 | 0.18 | 12/4/20 | PAR | RS003 | 31.7 | 0.44 | 4/23/22 | VIE |
| JG052 | 41.8 | 0.96 | 12/4/20 | PAR | RS005 | 47 | 1.35 | 4/23/22 | VIE |
| LR003 | 25.4 | 0.260 | 8/6/20 | BDS | RS007 | 35.1 | 0.52 | 4/23/22 | VIE |
| LR007 | 35.6 | 0.668 | 8/6/20 | BDS | RS008 | 35.7 | 0.56 | 4/23/22 | VIE |
| LR012 | 43.7 | 1.186 | 8/6/20 | BDS | RS009 | 32.6 | 0.44 | 4/23/22 | VIE |
| LR022 | 36.8 | 0.730 | 8/6/20 | MAY | RS010 | 38.5 | 0.7 | 4/23/22 | VIE |
| LR024 | 26.7 | 0.290 | 8/6/20 | MAY | RS011 | 28.5 | 0.29 | 4/23/22 | VIE |
| LR037 | 43 | 0.9 | 8/19/20 | ANA | RS012 | 36.9 | 0.6 | 4/23/22 | VIE |
| LR040 | 39.5 | 0.9 | 8/19/20 | ANA | RS013 | 26.8 | 0.23 | 4/23/22 | VIE |
| LR045 | 43.1 | 1.1 | 8/19/20 | ANA |  |  |  |  |  |

Table S1. Sample information for the queen snappers (*Etelis oculatus*) in this study. Missing values are identified by (-). GUA = Guánica, CDM = Caja de Muertos, PAR = La Parguera, BDS = Bajo de Sico, MAY = Mayagüez, ANA = Añasco, SJU = San Juan, VIE = Vieques.

| **File** | **Total** | **Low Quality** | **Ambiguous RAD-Tag** | **Retained Reads** | **Retention (%)** |
| --- | --- | --- | --- | --- | --- |
| JG009 | 8032422 | 2632 | 97740 | 7932050 | 98.75 |
| JG013 | 7752450 | 2614 | 33580 | 7716256 | 99.53 |
| JG018 | 8465396 | 2699 | 7674 | 8455023 | 99.88 |
| JG019 | 9442938 | 3118 | 48195 | 9391625 | 99.46 |
| JG021 | 8955714 | 2915 | 36427 | 8916372 | 99.56 |
| JG023 | 9126462 | 2731 | 16522 | 9107209 | 99.79 |
| JG034 | 9787918 | 3161 | 15201 | 9769556 | 99.81 |
| JG035 | 8859606 | 2597 | 22197 | 8834812 | 99.72 |
| JG037 | 8846322 | 2814 | 18511 | 8824997 | 99.76 |
| JG048 | 8970094 | 2980 | 23573 | 8943541 | 99.70 |
| JG052 | 9479156 | 3101 | 7315 | 9468740 | 99.89 |
| LR003 | 7313670 | 2259 | 24095 | 7287316 | 99.64 |
| LR007 | 7433526 | 2394 | 52334 | 7378798 | 99.26 |
| LR012 | 7466994 | 2437 | 35283 | 7429274 | 99.49 |
| LR022 | 7767456 | 2509 | 10277 | 7754670 | 99.84 |
| LR024 | 8815618 | 2855 | 69165 | 8743598 | 99.18 |
| LR037 | 9148002 | 2929 | 54861 | 9090212 | 99.37 |
| LR040 | 8720070 | 2842 | 14647 | 8702581 | 99.80 |
| LR045 | 9279046 | 2965 | 13581 | 9262500 | 99.82 |
| LR062 | 8698984 | 2711 | 12903 | 8683370 | 99.82 |
| RA001 | 8390952 | 2775 | 13302 | 8374875 | 99.81 |
| RA002 | 8066042 | 2645 | 14807 | 8048590 | 99.78 |
| RA003 | 6882334 | 2302 | 6034 | 6873998 | 99.88 |
| RA004 | 6888518 | 2055 | 31052 | 6855411 | 99.52 |
| RA005 | 5706644 | 1909 | 30055 | 5674680 | 99.44 |
| RA006 | 7324384 | 2344 | 43516 | 7278524 | 99.37 |
| RS001 | 7177884 | 2322 | 28030 | 7147532 | 99.58 |
| RS002 | 7860728 | 2483 | 21503 | 7836742 | 99.69 |
| RS003 | 7906894 | 2537 | 7108 | 7897249 | 99.88 |
| RS005 | 5644902 | 1768 | 224011 | 5419123 | 96.00 |
| RS007 | 6535676 | 2124 | 10408 | 6523144 | 99.81 |
| RS008 | 7717010 | 2500 | 8818 | 7705692 | 99.85 |
| RS009 | 7103738 | 1182 | 9970 | 7092586 | 99.84 |
| RS010 | 7284188 | 2360 | 54749 | 7227079 | 99.22 |
| RS011 | 7212678 | 2360 | 19305 | 7191013 | 99.70 |
| RS012 | 8268312 | 2705 | 3310 | 8262297 | 99.93 |
| RS013 | 6765292 | 2066 | 18292 | 6744934 | 99.70 |
| **Total** | 295098020 | 93700 | 1158351 | 293845969 | 99.54 |

Table S2. Table of the *process_radtags* reads results per queen snapper (*E.oculatus*).

| **M and n value** | **Number of R80 Loci** | **Change of R80 Loci** |
| --- | --- | --- |
|  |  |  |
| 1 | 48841 | - |
| 2 | 48953 | 112 |
| 3 | 47398 | -1555 |
| 4 | 45081 | -2317 |
| 5 | 42570 | -2511 |
| 6 | 39987 | -2583 |
| 7 | 37589 | -2398 |
| 8 | 35659 | -1930 |
| 9 | 34109 | -1550 |
| 10 | 32979 | -1130 |
| 11 | 32148 | -831 |
| 12 | 31555 | -593 |
| 13 | 31180 | -375 |

Table S3. Number of loci built and the change in loci between the runs of *denovo_map.pl* for the optimization protocol with the subsamples of queen snapper (*E. oculatus*).

| **Sample** | **# of Loci** | **Mean coverage** | **# Unpaired Reads** | **# PCR duplicates** | **Rate of PCR duplicates** |
| --- | --- | --- | --- | --- | --- |
| JG009 | 67174 | 24.518 | 60320 | 1340958 | 0.449 |
| JG013 | 64832 | 25.365 | 61197 | 1323122 | 0.446 |
| JG018 | 65157 | 27.39 | 65670 | 1473630 | 0.452 |
| JG019 | 66112 | 29.581 | 75634 | 1641732 | 0.456 |
| JG021 | 65083 | 28.562 | 71017 | 1564647 | 0.457 |
| JG023 | 64752 | 29.469 | 74523 | 1593975 | 0.455 |
| JG034 | 64933 | 31.26 | 78922 | 1725045 | 0.459 |
| JG035 | 64722 | 28.521 | 70015 | 1554798 | 0.457 |
| JG037 | 64787 | 28.638 | 72190 | 1536142 | 0.453 |
| JG048 | 65248 | 28.659 | 73224 | 1548566 | 0.453 |
| JG052 | 65118 | 30.013 | 74717 | 1674447 | 0.461 |
| LR003 | 64375 | 24.005 | 58491 | 1264841 | 0.45 |
| LR007 | 63941 | 24.551 | 56895 | 1255641 | 0.444 |
| LR012 | 65081 | 24.353 | 59575 | 1255842 | 0.442 |
| LR022 | 64606 | 25.61 | 62301 | 1329911 | 0.446 |
| LR024 | 67020 | 26.038 | 66643 | 1421499 | 0.449 |
| LR037 | 66358 | 28.004 | 73008 | 1535247 | 0.452 |
| LR040 | 64750 | 28.387 | 69569 | 1536618 | 0.455 |
| LR045 | 65089 | 29.812 | 74629 | 1630253 | 0.457 |
| LR062 | 65193 | 28.391 | 71617 | 1533262 | 0.453 |
| RA001 | 64980 | 27.871 | 68641 | 1431722 | 0.442 |
| RA002 | 63812 | 27.038 | 65606 | 1394072 | 0.447 |
| RA003 | 64582 | 22.92 | 53445 | 1164899 | 0.44 |
| RA004 | 66799 | 21.273 | 51595 | 1134041 | 0.444 |
| RA005 | 63551 | 17.246 | 54188 | 1146641 | 0.511 |
| RA006 | 64681 | 21.694 | 66856 | 1459819 | 0.51 |
| RS001 | 64016 | 21.686 | 69141 | 1434034 | 0.508 |
| RS002 | 65857 | 22.968 | 75523 | 1580333 | 0.511 |
| RS003 | 63490 | 24.183 | 76575 | 1588732 | 0.509 |
| RS005 | 62391 | 16.439 | 48470 | 1022970 | 0.499 |
| RS007 | 62602 | 20.38 | 59921 | 1305274 | 0.506 |
| RS008 | 64454 | 22.44 | 66222 | 1494891 | 0.508 |
| RS009 | 64830 | 29.662 | 92802 | 864538 | 0.31 |
| RS010 | 65359 | 21.352 | 67724 | 1452142 | 0.51 |
| RS011 | 64632 | 21.969 | 68161 | 1461385 | 0.507 |
| RS012 | 64515 | 36.071 | 71335 | 989831 | 0.298 |
| RS013 | 63830 | 20.95 | 62423 | 1381684 | 0.508 |
| **Total** | 2454223 | 24.92 | 2373707 | 51709412 | 0.459 |

Table S4. Table with the number, coverage, filtered reads and PCR duplication rate from the loci built in *gstacks* per queen snapper (*E. oculatus*) sample.

Figure S1. BayeScan plot of 16,188 SNPs from queen snapper (*E. oculatus*) after 50,000 iterations.

Figure S2. Mean LnP(K) (±SD) over 10 runs for each K value. Plot was generated in StructureSelector (Li and Liu 2018).

|  | **BDS** | **MAY** | **ANA** | **PAR** | **GUA** | **CDM** | **SJU** | **VIE** |
| --- | --- | --- | --- | --- | --- | --- | --- | --- |
| **BDS** | **0.6969(0.0278)** | 0.0301(0.0274) | 0.0303(0.0277) | 0.0302(0.0276) | 0.0301(0.0277) | 0.0300(0.0275) | 0.0302(0.0279) | 0.1221(0.0466) |
| **MAY** | 0.0331(0.0300) | **0.6999(0.0300)** | 0.0334(0.0303) | 0.0332(0.0301) | 0.0332(0.0300) | 0.0334(0.0303) | 0.0332(0.0300) | 0.1005(0.0462) |
| **ANA** | 0.0281(0.0257) | 0.0281(0.0258) | **0.6947(0.0257)** | 0.0278(0.0256) | 0.0280(0.0257) | 0.0281(0.0258) | 0.0279(0.0256) | 0.1373(0.0454) |
| **PAR** | 0.0255(0.0234) | 0.0256(0.0236) | 0.0259(0.0239) | **0.6925(0.0239)** | 0.0254(0.0234) | 0.0257(0.0239) | 0.0256(0.0236) | 0.1539(0.0442) |
| **GUA** | 0.0304(0.0279) | 0.0305(0.0278) | 0.0303(0.0275) | 0.0303(0.0276) | **0.6970(0.0277)** | 0.0303(0.0277) | 0.0300(0.0275) | 0.1213(0.0462) |
| **CDM** | 0.0305(0.0280) | 0.0305(0.0279) | 0.0307(0.0278) | 0.0304(0.0276) | 0.0304(0.0276) | **0.6974(0.0281)** | 0.0305(0.0276) | 0.1196(0.0462) |
| **SJU** | 0.0236(0.0221) | 0.0238(0.0221) | 0.0238(0.0224) | 0.0236(0.0220) | 0.0237(0.0221) | 0.0237(0.0220) | **0.6905(0.0222)** | 0.1674(0.0429) |
| **VIE** | 0.0174(0.0167) | 0.0176(0.0167) | 0.0178(0.0168) | 0.0173(0.0165) | 0.0175(0.0167) | 0.0176(0.0168) | 0.0350(0.0227) | **0.8597(0.0369)** |

Table S5. Inferred (posterior mean) migration rates of *E. oculatus* per sites. The source populations are represented by columns, whereas the receiving populations are represented by the rows. Bold rates indicates the self-recruitment at each site.
